# Low Testosterone in Adolescents & Young Adults

**DOI:** 10.3389/fendo.2019.00916

**Published:** 2020-01-10

**Authors:** Jordan Cohen, Daniel E. Nassau, Premal Patel, Ranjith Ramasamy

**Affiliations:** ^1^Department of Urology, University of Miami Miller School of Medicine, Miami, FL, United States; ^2^Department of Urology, Lenox Hill Hospital, Donald and Barbara Zucker School of Medicine at Hofstra/Northwell, New York, NY, United States

**Keywords:** testosterone, obesity, diabetes, adolescence, fertility

## Abstract

Male hypogonadism, the clinical syndrome with variable symptoms associated with gonadal dysfunction, can affect men of all ages. In older males, physiologic changes of the aging testis, account for the majority of decreased testosterone levels in this population. For younger males and adolescents, the etiology of hypogonadism is commonly due to congenital or acquired conditions that disrupt the testis production of testosterone or signaling from the hypothalamic-pituitary-gonadal axis. Diagnosis of hypogonadism in younger males can be a challenge, as symptoms such as decreased libido or erectile dysfunction, common in the older men, are not usually present, and young men instead commonly complain of low energy. While an underlying congenital cause should always be considered in young men with hypogonadism, acquired conditions such as obesity, diabetes, anabolic steroid or illicit drug use have all been associated with low testosterone levels. Outside of modifying identifiable risk factors for hypogonadism, pharmacologic testosterone therapy can also lead to therapeutic dilemmas in young men who desire paternity. Topical or injectable administration of testosterone, through negative feedback on the hypothalamus and pituitary, can decrease spermatogenesis, posing an infertility risk. Other agents that can replace testosterone or increase the body's natural production of testosterone without decreasing spermatogenesis are preferred, such as intranasal testosterone, selective estrogen modulators, aromatase inhibitors or human-chorionic gonadotrophin, often used in combination. Clinicians must maintain a high level of suspicion to properly diagnose young men with hypogonadism and tailor treatment based on both the underlying etiology and fertility goals.

## Introduction

Ninety-five percent of the serum testosterone (T) in males is synthesized by the Leydig cells of the testis under the influence of luteinizing hormone (LH) secreted from the pituitary gland. Defects, whether acquired or congenital, that interfere with the testis production of T or interactions with the hypothalamic-pituitary-gonadal axis (HPGA) can cause decreased T. Male hypogonadism is the clinical condition representing a constellation of symptoms along with gonadal dysfunction of either Leydig cells leading to decreased T or Sertoli cell/germ cells and subsequent decreased sperm production, often occurring together. The normal range for morning T in a male is between 300 and 1,000 ng/dl and likewise, hypogonadism has been defined as total T < 300 ng/dL by the Endocrine Society clinical practice guidelines (1, 2).

Hypoandrogenism is a common diagnosis in older men because the aging testis loses its ability to produce adequate levels of T despite normal or unchanged levels of LH (3). Approximately 40% of men over the age of 45 and 50% of men in their 80s are hypogonadal (4, 5). T levels have been found to decrease by 100 ng/dL every 10 years or an average rate of 1–2% per year beyond age 30, which may further vary by adiposity, medications, and chronic disease (6–8). In the elderly population, symptoms highly suggestive of hypogonadism include decreased spontaneous erections, decreased nocturnal penile tumescence, decreased libido, and reduced testicular volume (9).

Given that hypogonadism has been defined more so based on adults, in children and adolescents, a distinction should be made. If not occurring together, in hypogonadism as hypoandrogenism (Leydig cell dysfunction), then other testicular cell populations, germ cell and/or Sertoli cells, should be considered for possible dysfunction. Androgen and gonadotropin levels in pre-pubertal children are generally low, and even those with primary gonadal failure may fall within the expected normal range for their age, further compounding diagnosis before puberty. Therefore, the assessment of Sertoli cells is essential to diagnose hypogonadism in a prepubertal population. It is estimated that ~70% of children with hypogonadism will be misdiagnosed if based on serum gonadotropin measurement (10). There is also increasing evidence that anti-Mullerian and inhibin B levels can improve the sensitivity and result in earlier diagnosis which ultimately allows for treatment to start at a younger age (10).

In younger men, the etiology of hypogonadism may be related to an underlying genetic condition, a primary problem with the HPGA, environmental factors or from past infection or injury to the testis (11). Diagnosis of hypoandrogenism in healthy adolescents can be challenging, as the symptoms that correlate with a decreased T level are different than in the elderly population. In a recent study, hypogonadal symptoms in men aged <40 years were associated with a total T level of <400 ng/dL. Of the hypogonadal symptoms evaluated with the Aging Male (ADAM) questionnaire, “lack of energy” appears to be the most important symptom that predicted a total T level of <400 ng/dL in the under 40 population as opposed to decreased libido and erectile dysfunction which were more frequent complaints in the elderly population (11). Additionally, one must carefully consider testosterone replacement therapy (TRT) in the younger population as it could interfere with spermatogenesis and fertility. This brief review will expand on the etiology, diagnosis, and treatment options for hypogonadism in the young adult male.

## Etiology of Hypogonadism in Adolescent and Young Adulthood Men

The HPGA is of paramount importance in the processes related to the development, maturation, and sustainability of male hormonal balance. The pulsatile secretion of gonadotropin-releasing hormone (GnRH) by the hypothalamus stimulates LH and follicle-stimulating hormone (FSH) production and secretion by the anterior pituitary gland. LH stimulates testosterone production from the interstitial Leydig cells of the testes. This is required for male internal and external reproductive organ development and later differentiation of secondary human sexual characteristics. FSH, in turn, sustains testicular function via Sertoli cells through spermatogenesis (12).

Many of the causes of hypoandrogenism in the adolescents may be transient, with resolution of the low androgen level once the underlying condition is resolved or improved. Table 1 lists the known congenital or acquired disorders at the testicular (primary hypogonadism) or HPGA (secondary hypogonadism) conditions resulting in androgen deficiency (12). Delayed puberty in adolescents is most often caused by constitutional delay of growth (>60%) whereas primary and secondary hypogonadism explains <20% of cases of delayed pubertal hypogonadism (11, 13). Primary hypogonadism (also known as hypergonadotropic hypogonadism) is caused by an inherent defect within the testes. This condition is biochemically characterized by low or absent testosterone levels and high gonadotropin levels. Spermatogenesis is usually severely impaired and not responsive to hormonal therapy. This is in contradistinction to secondary hypogonadism (also known as central hypogonadism or hypogonadotropic hypogonadism), which is caused by a dysfunction in the hypothalamus and/or the pituitary gland. This condition is biochemically characterized by low or inappropriately normal gonadotropins levels along with low total testosterone levels. Spermatogenesis is impaired but is usually responsive to hormonal therapy (14). A number of genetic loci (ANOS1, FGFR1, KISS1, KISS1R, TAC3) have been implicated in the development and migration of GnRH or the synthesis and secretion of GnRH itself (15). Beyond genetic causes, pediatric and pubertal hypogonadism may be seen in patients with chronic diseases and excessive drug usage.

**Table 1:** Causes of hypogonadism.

**Primary hypogonadism**	**Secondary hypogonadism**
**Congenital disorders**	
Klinefelter syndrome	Kallmann syndrome
Mutation in LH receptor genes	Prader-Willi syndrome
Mutation in FSH receptor genes	Congenital adrenal hyperplasia
Androgen synthesis disorders	Chronic systemic illness
Varicocele	Gonadotropin subunit mutation
Cryptorchidism	Pituicyte differentiation gene mutation
Myotonic dystrophy	Hyperprolactinemia
	Trauma
**Acquired Disorders**	Medications *(steroids, opiates)*
	Diabetes Mellitus
Infections (*mumps*)	Benign tumors
Radiation	Malignant tumors
Medications	Infiltrative diseases
*(alkylating agents, ketoconazole, glucocorticoids)*	Idiopathic
Testicular torsion	
Environmental toxins	
Chronic systemic illnesses (*HIV, renal failure)*	
Idiopathic	

Beyond the age of 30 there is a decline in the levels of both circulating total and free T. The difference between the decline of total T and free T during the aging process is explained by an age-related increase in circulating concentration of sex hormone-binding globulin (SHBG), which reduces the proportion of free T (16, 17). In healthy men, the age-related decline of T coupled with an increase in LH, supports a diagnosis of primary testicular failure compensated for by an increased LH secretion. The age-related decrease in T reflects general age-related cellular degeneration, reduced number of functional Leydig cells and atherosclerosis of testicular arterioles (18). For the vast majority of men, T levels within the normal young adult reference ranges in the absence of hypogonadal symptoms is clinically irrelevant. However, in the developing world a number of increasing conditions may be shifting the prevalence of hypogonadism to a younger age including diabetes, obesity, and rising rates of opioid use (19).

The prevalence of obesity in young adults is increasing at a staggering rate and is anticipated to triple within the next decade (20). A study looking at the Adolescent and Young population utilizing the National Health and Nutrition Examination Surveys (NHANES) demonstrated a statistically significant increase in BMI from 1999 to 2016. In the European Male Aging Study, 73% of men with reduced testosterone were overweight or obese and serum T in men with a BMI > 30 kg/m^2^ was on average 5 nmol/l lower than those with normal weight (21). The obesity related decline of T levels is multi-factorial and can be associated with a decrease in SHBG and/or an increased conversion of T to estrogen by peripheral adipose tissue (11). Along with declining serum testosterone levels, a large meta-regression analysis has demonstrated a significant decline in sperm counts between 1973 and 2011 (22). The cause of this substantial decline has yet to be fully elucidated, but pituitary inhibition causing testosterone deficiency and thus, a decrease in sperm counts requires further investigation.

Further compounding the underlying cause of hypogonadism in young obese men is the possibility of a concurrent diagnosis of type 2 diabetes mellitus, which has been increasing at a yearly rate of 4.8% compared to 1.8% for type 1 (23). The rate of new diagnosed cases of type II diabetes in 10–19 year olds rose most sharply in Native Americans (8.9%) an, Asian Americans/Pacific Islanders (8.5%) and non-Hispanic blacks (6.3%) (23). A recent study by Chosich et al. found that hyperinsulinemia, in conjunction with elevated serum lipid levels, suppresses pituitary gonadotropin release, providing a mechanistic explanation for decreased androgen levels in this cadre of patients (24). With the rising epidemic of adolescent and young adult obesity and type II diabetes, it is plausible that these conditions in isolation or in tandem may explain lower than normal androgen levels in patients aged twenty to forty. The rising incidence of obesity and diabetes in the young adult population is a major expenditure of health care dollars and while the low androgen levels are a secondary observation it is critical that proactive measures by primary care physicians and community wellness fairs address this global issue.

Another increasing cause of androgen deficiency at a population health level has been from the staggering rise in illicit drug use. Nearly 2 million people in the United States suffer from substance abuse disorders with 47,000 dying annually from opioid overdose including prescription opioids, fentanyl, and heroin (25). Opioid-induced androgen deficiency has also risen dramatically in the last 10–15 years. Sexual dysfunction is reported in 85% of heroin addicts and 81% on a stable methadone maintenance regimen, although this might be due to additional factors (26). Chronic opioid use disrupts the HPGA creating a secondary hypogonadism. Opioid receptors μ (MOR), δ (DOR), and κ (KOR) are present in the hypothalamus and the pituitary with activation leading to a suppressed HPGA and subsequent decrease in serum T within hours of opioid administration (27). The individual opioid drugs have been shown to affect the clinical symptoms associated with low androgen levels differently. Patients taking hydrocodone and hydromorphone were least affected by clinical androgen deficiency as opposed to men taking fentanyl or morphine where the incidence was much higher (27). Although not opioids, chronic ingestion of analgesics such as acetaminophen, acetylsalicylic acid, and ibuprofen, have also been shown to alter testicular physiology. In a randomized control trial involving young men taking ibuprofen, the free T/LH ratio declined by 18% after 2 weeks compared with the placebo group. The ibuprofen cohort also exhibited a decline in anti-Müllerian hormoe indicating that both the Leydig and Sertoli cell lines were negatively affected (28).

Anabolic steroids are another class of agents causing androgen deficiency. Anabolic steroids suppress endogenous T production via negative feedback on the HPGA resulting in a concurrent diminution in testicular size and low sperm count. In a recent meta-analysis of thirty-three studies with 3,879 participants (1,766 anabolic androgen steroid users and 2,113 non-anabolic androgen users) the anabolic users showed a significant reduction in LH (weighted mean difference −5.05 to −1.07) and endogenous T levels (weighted mean difference −10.75 nmol/L to −15.01 nmol/L, *p* < 0.001) (29). After drug discontinuation the gonadotropin levels returned to normal within 13–24 weeks; however, serum T levels remained reduced beyond 4 months (29). Young adults presenting with clinical symptoms suggestive of androgen deficiency should be queried for use/abuse of anabolic androgen steroid usage.

Exposure to several environmental toxins may also contribute to hypogonadism, notably Sertoli and germ cell dysfunction. Tobacco smoke contains highly carcinogenic nitrosamines, polycyclic aromatic hydrocarbons (benzopyrene), and volatile organic compounds (benzene) (30). Increased seminal levels of reactive oxygen species from smoking impair sperm function and data shows that nicotine and its metabolites are capable of crossing the blood-testis barrier (31). Separately, pesticides and herbicides result in high serum levels of polychlorinated biphenyls. Men in the highest quartile of consumption of high pesticide-residue fruit and vegetables (≥1.5 servings/day) had a 49% lower total sperm count compared to men in the lowest quartile (<0.5 servings/day) (32). Heavy metals like mercury and lead have also been correlated to male infertility (33). Along with the aforementioned etiologies of hypogonadism, this highlights the importance of a comprehensive patient history in order to elucidate underlying factors that may contribute to hypogonadism in younger patients.

## Diagnosis

The clinical symptoms of hypogonadism are non-specific, making diagnosis challenging in adolescents and young men. Given the numerous pathways within the HPGA and the slow changes to the hormonal levels, the signs and symptoms suggestive of androgen deficiency take time to clinically manifest. The signs and symptoms of low androgen levels include reduced sexual desire and activity, erectile dysfunction, decreased spontaneous erections, incomplete or delayed sexual development, small testes, gynecomastia, loss of body hair/reduced shaving, subfertility, and reduced bone mass. Less specific symptoms and signs are decreased energy and motivation, reduced physical performance, depressed mood, poor concentration and memory, sleep disturbances, anemia, reduced muscle mass, and increased body fat (8).

Ultimately, the diagnosis can be made by a obtaining a fasting serum T level between 7:00 and 11:00 AM, or within 3 h of waking up. While consensus lacks for the exact biochemical level at which to ascribe the diagnosis of hypogonadism, a recent publication by the Endocrine Society with support from the US Center for Disease Control has a level < 264 ng/dl in nonobese males as diagnostic for androgen deficiency (34). This threshold along with various others defining low total T set from 250 to 300 ng/dl by other societies such as the American Urological Association have been established regardless of age following many large-scale population studies (35). According to the European Male Aging Study at least three clinical sexual symptoms should be present in conjunction with the laboratory abnormal values to confirm the diagnosis of androgen deficiency (8). As mentioned previously, symptoms of fatigue and lack of energy may be more specific in the younger adult cohort than sexual symptoms. Following confirmation of low serum T levels and concomitant signs and symptoms of hypogonadism, clinicians should use serum LH and FSH in conjunction with testosterone to differentiate between primary and secondary hypogonadism.

## Treatment Options for Adolescent & Young Adulthood Men With Low T

When designing a treatment plan for TRT in young adults, clinicians must understand that most exogenous T therapy will suppress spermatogenesis and decrease fertility potential (36, 37). As such, identifying modifiable risk factors that may lead to hypogonadism should be an early step in patient evaluation as correction of many of the aforementioned conditions may mitigate the need for TRT. Patients with obesity, poorly controlled diabetes, or opioid usage should be counseled on weight loss, diet, exercise, and drug abuse before starting testosterone replacement therapy (TRT). Formal weight loss programs have not only shown that the percentage of weight loss correlated with increased T levels, but parameters related to fertility including sperm motility and morphology also improved (38). While there is evidence that T may reduce HbA_1C_, lower BMI, and reduce waist circumference other studies have not shown any change in HbA_1C_ with testosterone compared to placebo (39).

Non-exogenous TRT therapy aims to either increase the body's production of T or decrease the conversion of T to estrogen in adipose tissue. HCG, a chemically similar hormone to LH, can be administered parenterally to stimulate Leydig cell production of T, while maintaining intratesticular T needed for spermatogenesis (40). Clomiphene citrate, a selective estrogen receptor modulator, binds receptors on the hypothalamus and pituitary gland to reduce estrogen's negative feedback on the HPGA, thereby increasing production of GnRH, LH and FSH. The increase in gonadotropic hormones then results in increased testosterone production in the testes (41). Adipose tissue, especially in obese individuals, contains aromatase which converts T into estrogen. Anastrazole, an aromatase inhibitor, is also employed to increase T levels and is beneficial for spermatogenesis if the serum T to estrogen ratio is < 10 (41, 42). While these medications are efficacious, using them for male hypogonadism treatment is considered off-label by the Food and Drug administration and carry side effects of decreased bone mineral density and libido (42).

Short acting, T nasal gel (Natesto®) is an exogenous T that may not decrease spermatogenesis. Early results from a phase 4 trial reported that Natesto significantly increased median AM T levels without affecting median FSH, LH and semen parameters at 6 months follow-up. It is postulated that the short half-life of intranasal T maintains the pulsatile release of GnRH compared to other forms of exogenous T therapy which negatively impact the HGPA and therefore prevent the steep decline in LH and FSH to maintain spermatogenesis. Further benefit of Natesto compared to other forms of exogenous TRT includes the ease of delivery, no need for needles and decreased risk of transference (43).

With the availability of new T formulations in combination with aggressive consumer advertising there has been an exponential rise in the use of TRT for late onset hypogonadism. In the USA, sales of T preparations quadrupled between 2000 and 2011, although the number of low serum T in commercial laboratories has remained relatively constant (44). This suggests that commercially available T replacement is being used for symptoms such as decreased libido, erectile dysfunction depression, and fatigue who may in fact have other clinical conditions rather than androgen deficiency itself.

T therapy is associated with adverse effects on the cardiovascular and hematologic systems and caution should be exercised when prescribing testosterone for clinical symptoms without truly confirmed biochemical abnormalities per guidelines (45). Although to date there is no knowledge on the specific use of testosterone preparations in the younger adult cohort, it is likely that this group may also be using the formulations for symptoms and signs suggestive, but not documented of androgen deficiency.

## Conclusion

Male adolescents may present with few typical signs of adult hypogonadism and biochemical androgen levels need to be followed judiciously in the adolescent and young adult cohort so that effective strategies based on clinical research can be recommended. However, for young adult patients <30 years of age who truly have hypoandrogenism with serum total T <300–400 ng/dl there needs to be randomized clinical trials establishing the safety and efficacy of long-term TRT. Hypogonadism is a cluster of numerous conditions and a single therapy cannot be the same for all. At a population health level, beginning in adolescence and moving through young adulthood there needs to be concerted efforts to improve overall cardiovascular health, reduce type II diabetes, decrease the rising incidence of obesity especially in the African-American, Hispanic and Native American populations, and stem the escalating opioid addiction our country faces. Future studies, especially in the young adult population, need to systemically evaluate these patients and improve outcomes for tailored treatment regimens based on underlying cause and paternity preferences.

## Author Contributions

JC: primary manuscript writer and conducted an analysis of background literature. DN: secondary manuscript writer and helped with background literature. PP: wrote specific sections. RR: main outlining, final edits, writing, and organization.

### Conflict of Interest

RR is an investigator for Aytu Biosciences, the manufacturer of Natesto. The remaining authors declare that the research was conducted in the absence of any commercial or financial relationships that could be construed as a potential conflict of interest.
