# Corrigendum: Low Testosterone in Adolescents & Young Adults

**DOI:** 10.3389/fendo.2020.00449

**Published:** 2020-07-14

**Authors:** Jordan Cohen, Daniel E. Nassau, Premal Patel, Ranjith Ramasamy

**Affiliations:** ^1^Department of Urology, University of Miami Miller School of Medicine, Miami, FL, United States; ^2^Department of Urology, Lenox Hill Hospital, Donald and Barbara Zucker School of Medicine at Hofstra/Northwell, New York, NY, United States

**Keywords:** testosterone, obesity, diabetes, adolescence, fertility

In the original article, we neglected to state a commercial relationship. A correction has been made to ***Conflict of Interest:*** “RR is an investigator for Aytu Biosciences, the manufacturer of Natesto.” The corrected Conflict of Interest statement appears below. The authors apologize for this error and state that this does not change the scientific conclusions of the article in any way. The original article has been updated.

## Conflict of Interest

RR is an investigator for Aytu Biosciences, the manufacturer of Natesto. The remaining authors declare that the research was conducted in the absence of any commercial or financial relationships that could be construed as a potential conflict of interest.

